# Solubility and Stability of Carotenoids in Ammonium- and Phosphonium-Based Ionic Liquids: Effect of Solvent Nature, Temperature and Water

**DOI:** 10.3390/molecules28083618

**Published:** 2023-04-21

**Authors:** Wanting Cheng, Feng Xian, Zhanluo Zhou, Kun Hu, Jing Gao

**Affiliations:** 1 Collage of Food Science, Guangdong Pharmaceutical University, Guangzhou 510006, China; 2 Collage of Food Science and Technology, Guangdong Ocean University, Zhanjiang 524091, China

**Keywords:** ionic liquid, carotenoid, physicochemical property, solubility, stability

## Abstract

Ionic liquids (ILs) have arisen as alternatives to organic solvents and been used in natural pigment extraction in recent decades. However, the solubility and stability of carotenoids in phosphonium- and ammonium-based ILs are insufficiently explored. In this work, the physicochemical properties of the ILs, and the dissolution behavior and storage stability of three carotenoids (astaxanthin, β-carotene, and lutein) in the IL aqueous solution were investigated. The results showed that the solubility of the carotenoids in the acidic IL solution is higher than that in the alkaline IL solution, and the optimal pH is about 6. The solubility of astaxanthin (40 mg/100 g), β-carotene (105 mg/100 g), and lutein (5250 mg/100 g) was the highest in tributyloctylphosphonium chloride ([P_4448_]Cl) due to the van der Waals forces with [P_4448_]^+^ and hydrogen bonding with Cl^−^. A high temperature was beneficial to improve the solubility, but it can reduce the storage stability. Water has no significant effect on the carotenoid stability, but a high water content decreases the carotenoid solubility. A IL water content of 10–20%, an extraction temperature of 338.15 K, and a storage temperature of less than 298.15 K are beneficial for reducing the IL viscosity, improving carotenoid solubility, and maintaining good stability. Moreover, a linear correlation was found between the color parameters and carotenoid contents. This study provides some guidance for screening suitable solvents for carotenoid extraction and storage.

## 1. Introduction

Carotenoids are liposoluble compounds containing 40 carbon atoms with conjugated double bonds [1]. Carotenoids are divided into two main groups: carotenes and xanthophylls. Carotenes consist of hydrocarbon chains (e.g., α-carotene, β-carotene, and lycopene), and xanthophylls consist of oxygen as a functional group (e.g., astaxanthin, zeaxanthin, and lutein) [2,3]. Carotenoids have been widely used as colorants, antioxidants, and anti-inflammatory agents in different areas, such as the pharmaceutical [4,5], cosmeceutical [6], food [7,8], and feed [9,10] industries. The antioxidant capacity of astaxanthin is the highest [11], and lutein minimizes macular degeneration [12,13]. Recently, increasing attention has been paid to the extraction of natural carotenoids from plant resources. Astaxanthin of 19.8% was extracted from *Haematococcus pluvialis* by using hydrochloric acid pretreatment followed by acetone extraction [14]. Carotenoids were effectively extracted by anhydrous acetone from persimmon peel and pulp at 40 °C, and the yields of β-cryptoxanthin and β-carotene reached 16,709.90 mg/100 g and 4479.07 mg/100 g, respectively [15]. However, organic solvent extraction methods have huge disadvantages, such as their volatility and unknown biological toxicity. Therefore, the creation of new extraction solvents for carotenoids remains an important challenge, despite considerable progress.

A promising alternative is the use of ionic liquids (ILs). ILs are molten salts formed by organic cations and organic or inorganic anions and have been widely used as solvents in the separation and purification of bioactive compounds [16,17,18]. The most attractive characteristics of ILs are their negligible vapor pressure, improved solvation ability, non-flammability, and high ionic conductivity and solubility. In a previous study, 1-hexyl-3-methylimidazolium acetate ([C_6_mim][OAc]) improved the yields of zeaxanthin by 23.08% in comparison to a mixture of hexane, acetone, and ethanol [19]. The extraction yield of lutein from Chlorella reached 5.6 mg/g when using 1-butyl-3-methylimidazolium bromide ([C_4_mim]Br), which was higher than when using ether (4.7 mg/g) [20]. It should be emphasized that most studies have focused on imidazolium-based ILs. In fact, the ILs with higher shielded charges, such as tetrabutylammonium chloride ([N_4444_]Cl) and tetrabutylphosphonium chloride ([P_4444_]Cl), show higher hydrophobicity and are more suitable for the extraction of hydrophobic biomolecules. It was reported that the solubility of curcumin can reach nearly 55 mg/mL in aqueous solutions of tetradecyl triethyl ammonium bromide ([N_22214_]Br), which is greater than its solubility in 1-octy-3-methylimidazolium bromide ([C_12_min]Br) (15 mg/mL) [21]. However, limitations of the study include the lack of data concerning the carotenoid dissolution behavior and stability in ammonium- and phosphonium-based ILs, which is the main potential cause of insufficiency in the extraction of natural carotenoids.

Bi et al. [22] reported that viscosity, polarity, and physicochemical interactions play an important role in solid–liquid extraction. For example, the high viscosity of ILs limits the mass transfer between the extractive target and the extraction phase. The physicochemical properties of ILs are mainly determined by hydrogen bonding, van der Waals forces, molecular weight, and mobility, which can be adjusted by changing the type of anion and/or cation, temperature, and water content [23]. Most mixtures of ILs and water were highly temperature dependent. A binary system composed of tetrabutylphosphonium trifluoroacetate ([P_4444_]CF_3_COO) (50%) and water (50%) was a homogeneous mixture at 293.15 K but separated into two phases when the temperature increased above 303.15 K [24]. However, some ammonium-based ILs, such as betainium bis(trifluoromethylsulfonyl) amide ([Hbet][Tf_2_N]), showed “high-temperature homogenization and low-temperature separation” properties [25]. Moreover, water was always used as the co-solvent to decrease the viscosity of ILs and the extraction cost [26]. However, an excessive amount of water could cause damage to the supramolecular structure of the IL and lead to a significant weakening of interactions between the IL and the extractive target. For example, the extraction rate of astaxanthin extracted by 1-ethyl-3-methylimidazolium dibutyl phosphate ([C_2_mim]DBP) decreased from 18% to 2% when the content of the water in the IL increased from 20% to 80% [25]. Therefore, to select suitable ILs for carotenoid extraction, the anion/cation structure, temperature, and water content data should be considered. 

Despite numerous published papers devoted to investigating the effect of temperature and water on the properties of ILs, some details remain unclear for ammonium- and phosphate-based ILs, and existing studies on carotenoid extraction/analysis have not been reported. On the other hand, most pigments differ in solubility, color, sensitivity to environmental conditions, and stability in various solutions. Therefore, ammonium- and phosphate-based ILs were used as potential solvents for carotenoids in this study. The physicochemical properties of the ILs at different temperatures and water contents were investigated first. Then, the solubility of astaxanthin, lutein, and β-carotene in the ILs was measured, and the interaction between the carotenoid and IL was analyzed based on molecular simulation. Moreover, the color change and carotenoid degradation were determined to explore the stability of astaxanthin, lutein, and β-carotene in the IL systems. Finally, the relationship between the concentration of carotenoids and the chromatic parameters of IL aqueous solutions was calculated. This study aims to give guidance for screening suitable ILs for carotenoid extraction and applications.

## 2. Results and Discussion

### 2.1. Phase Behavior of IL–Water Systems

Compared with imidazolium-based ILs, ammonium- and phosphate-based ILs with higher shielded charges are presented in the literature as hydrophobic ILs, with some of them having a relatively high solubility in water. Herein, the phase behavior of the IL–water systems was measured at 298.15 K, and the apparent phase state for the IL–water systems is noted in Table 1. It can be seen that [N_4444_]Br, [N_4444_]Cl, [N_4444_]CF_3_COO, [P_4444_]Br, [P_4444_]Cl, [P_4444_]CF_3_COO, and [P_4448_]Cl formed a homogeneous phase when the water content increased from 10% to 50%, while [P_4448_]Br and [P_4448_]CF_3_COO were insoluble when the water content exceeded 20%. In solid–liquid extraction, the solvent should maintain its stability at different temperatures. Therefore, the homogeneous phase of the IL–water mixtures was selected as the preliminary solvent system.

### 2.2. Physicochemical Properties of IL Aqueous Solutions

**Figure 1:**
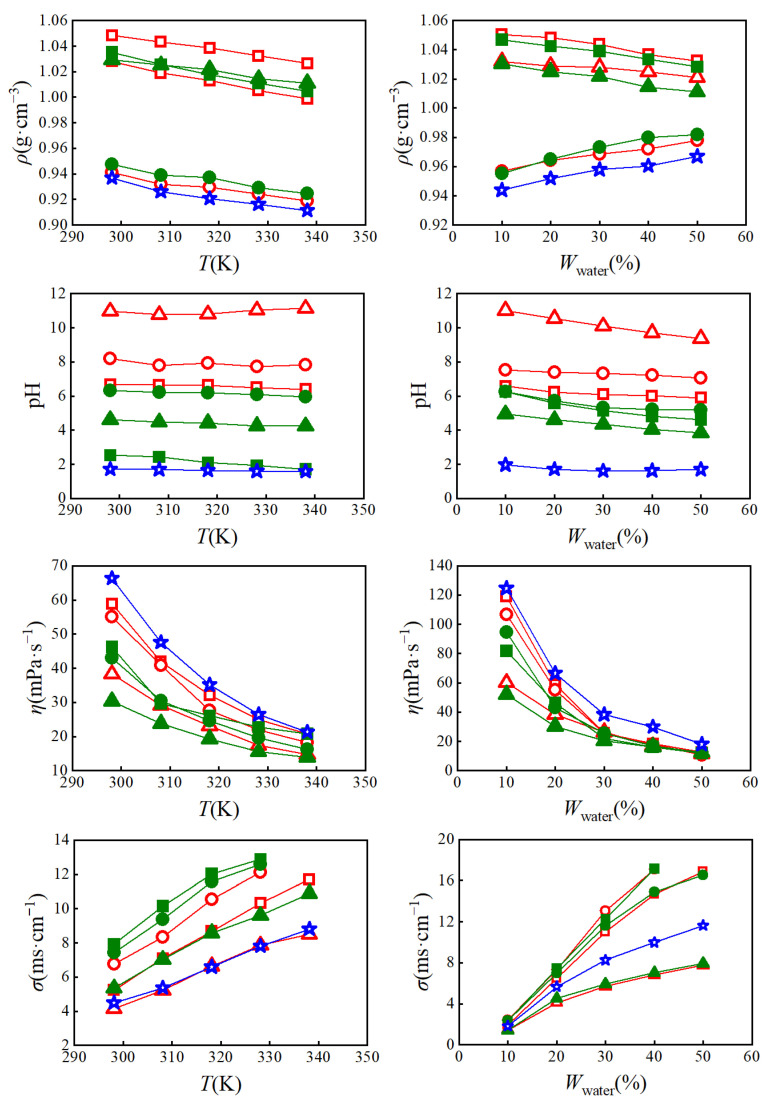
Effect of temperature (**left**, *w_water_* = 20%) and water content (**right**, *T* = 298.15 K) on the density, pH, conductivity, and viscosity of the IL aqueous solutions: (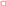) [N_4444_]Br, (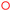) [N_4444_]Cl, (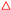) [N_4444_]CF_3_COO, (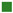) [P_4444_]Br, (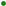) [P_4444_]Cl, (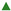) [P_4444_]CF_3_COO, (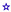) [P_4448_]Cl.

The data of the physicochemical properties (density, pH, viscosity, and conductivity) of the IL aqueous solutions at temperature from 298.15 K to 338.15 K in the presence of 10% to 50% water are summarized in Tables S1 and S2 and presented in Figure 1.

#### 2.2.1. Density

The density difference of the IL/water mixture is of great significance to the operation of separation equipment in industry. The density of ILs is mainly dependent on the void size and molar volume [27]. The extraction efficiency of ILs is highly dependent on their immiscibility with water based on the density difference. As shown in Table S1, the density of all the studied ILs lied in the range of 0.9113 to 1.0483 g·cm^−1^, which is a typical density range for ILs. It is worth noticing that the larger the cation radius of the IL, the lower its densities. For example, the densities of phosphonium-based ILs were smaller than ammonium-based ILs, and the density decreases with the increase in alkyl chain length. The densities of the ILs with different cations followed the trend [N_4444_]^+^ > [P_4444_]^+^ > [P_4448_]^+^, and the ILs containing different anions followed the trend Br^−^ > CF_3_COO^−^ > Cl^−^. Even though CF_3_COO^−^ has a higher molecular weight than Br^−^, the C=O bond in CF3COO^−^ combines with water to form strong hydrogen bonds, resulting in a decrease in density [28].

The molar volume and isotropic coefficient of expansion of the ILs increased with rising temperature (Table S1), because the kinetic energy of molecules increased at higher temperatures [29]. The densities of the IL/water mixture (*w_water_* = 20%) decreases linearly with the increase in temperature. Similarly, the density values of the 1-butyl-3-methylimidazolium tetrafluoroborate ([C_4_mim][BF_4_])/water mixture (*w_water_* = 0.0207, 0.0403, 0.0611 mol/kg) decreased with the rise in temperature from 293.15 K to 318.15 K [30]. Adding water resulted in a decrease in hydrogen bonds between molecules, which may be due to the reduced interaction as well as cation mobility. 

Generally, the densities of the studied ILs containing Cl^−1^ ([N_4444_]Cl, [P_4444_]Cl, and [P_4448_]Cl) were all smaller than 1.0 g·cm^−1^. The higher the water content in the IL, the higher the density of the mixtures. Therefore, the densities of [N_4444_]Cl, [P_4444_]Cl, and [P_4448_]Cl increased as the water content increased from 10% to 50%, while the densities of [N_4444_]Br, [P_4444_]Br, [N_4444_]CF_3_COO, and [P_4444_]CF_3_COO decreased to equal the density of water (Figure 1). 

#### 2.2.2. pH

The acidity and alkalinity of the extraction system is are important factors for selecting extraction equipment. In general, the studied IL aqueous solution presented a wide range of pH values [31], approximately ranging from 1 to 11. The ILs with [P_4444_]^+^ or [P_4448_]^+^ cations were acidic, while the ILs with [N_4444_]^+^ cations were neutral or basic. It is noteworthy that the properties of ILs may depend on the strength of the interaction between the anion and water [32]. CF3COO^−^ could form a relatively stable weak acid with H^−^ from H_2_O, and promoted the ionization of H_2_O. Therefore, the highest pH value of 11.13 was for the mixture of [N_4444_]CF_3_COO/water (*w_water_* = 20%, *T* = 338.15 K), and the lowest pH was 0.62 for the mixture of [P_4448_]Cl/water (*w_water_* = 10%, *T* = 298.15 K). 

#### 2.2.3. Viscosity

In green extraction technology, solvents with a low viscosity have a stronger advantage. Dannie et al. [33] suggested a viscosity of 100 mPa·s^−1^ as the maximum viscosity value in process engineering research. In recent years, imidazolium-based ILs have become widely used extraction solvents [18]. However, due to their high viscosity, cosolvents such as water or organic solvents were often added to enhance the mass transfer [34]. Królikowska et al. [35] reported that the viscosity of ethylsulfate-based ILs decreased with an increasing amount of water in binary mixtures. 

In this study, all viscosity values of ammonium- and phosphonium-based ILs (*w_water_* = 20%) were lower than 100 mPa·s^−1^ in the temperature range of 298.15–338.15 K. When the temperature was lower than 318.15 K or the water content was lower than 30%, the viscosities of the ILs with different anions and cations followed the trend [P_4448_]Cl > [N_4444_]Br > [N_4444_]Cl > [P_4444_]Br ≈ [P_4444_]Cl > [N_4444_]CF_3_COO > [P_4444_]CF_3_COO. When the temperature was increased to 328.15 K or the water content was more than 30%, the viscosity values of the ILs were very close to each other. The van der Waals forces and hydrogen bonding between the anions and cations of the ILs were weakened when increasing the temperature and adding water [36,37]. Therefore, the viscosity of all the ILs decreased significantly with the increase in water content from 10% to 30%, and the lower viscosity of approximately 15 mPa·s^−1^ could be obtained with a water content of 30% at 338.15 K.

#### 2.2.4. Conductivity

Conductivity, mainly related to the mobility of ions, is an essential parameter in evaluating aggregation behavior and the structure of solvents [38,39]. ILs with a higher viscosity exhibit a low conductivity. On the other hand, ILs with long alkyl chains have a lower conductivity than ILs with short alkyl chains, because the van der Waals force increases with the growth of the carbon chain [40]. Therefore, the conductivity of [P_4448_]Br (*w_water_* = 10%) was the lowest of 2 mS·cm^−1^ at 298.15 K. A higher temperature and water content resulted in a reduction in the intermolecular force between the anions and cations of the ILs, which enhanced the ionization and ion mobility. As shown in Figure 1, the conductivity of all IL aqueous solutions significantly increased with the increase in temperature and water content. The conductivity of [P_4448_]Br (*w_water_* = 10%) increased to 11.8 mS·cm^−1^ when the water content was 50%.

### 2.3. Dissolution Behavior of Carotenoids in IL Aqueous Solutions

#### 2.3.1. Solubility

**Figure 2:**
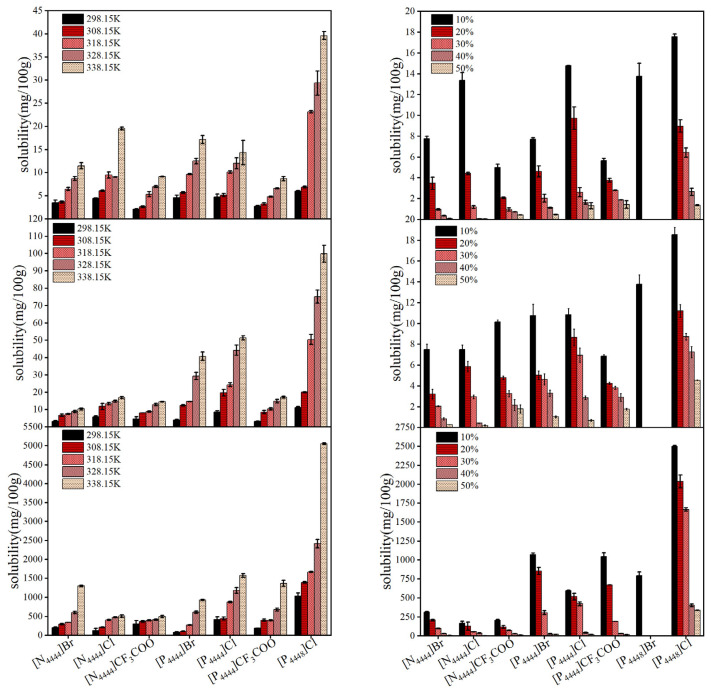
Solubility of astaxanthin (**top**), β-carotene (**middle**), and lutein (**bottom**) in the IL aqueous solutions as a function of temperature (**left**, *w_water_* = 20%) and water content (**right**, *T* = 298.15 K).

Solubility behavior is the most challenging aspect for carotenoid extraction. The solvation properties of phosphonium- and ammonium-based ILs strongly depend on their structure and miscibility with water and can be tailored by changing the temperature. Therefore, the solubilities of carotenoids in [N_4444_]Br, [N_4444_]Cl, [N_4444_]CF_3_COO, [P_4444_]Br, [P_4444_]Cl, [P_4444_]CF_3_COO, and [P_4448_]Cl were determined at temperatures from 298.15 to 338.15 K and water contents from 10% to 50%. The graphical results of solubility of astaxanthin, β-carotene, and lutein in IL aqueous solutions as a function of temperature (*w_water_* = 20%) and water content (*T* = 298.15 K) are presented in Figure 2. 

Generally, the solubility of the carotenoids in all studied ILs increased with the increase in temperature. The main reason may be that the viscosity of all ILs decreased significantly with the increase in temperature, as shown in Figure 1. On the other hand, the higher the water content in the ILs, the lower the viscosity. However, the solubility of the carotenoids decreased with the increase in water content. One potential explanation for the tendency might be that the anions and cations of the ILs were extremely solvated in excess water [41]. Similarly, the solubility of lamotrigine in aqueous mixtures of 1-octyl-3-methylimidazolium bromide IL decreased with the increasing concentration of water [42]. However, it should be stressed that pure ILs showed a very high viscosity, which is not conducive to extraction applications. For example, the viscosity of [P_4448_]Cl free of water at 298.15 K was 1260.0 mPa·s^−1^, which was 18.5 times the viscosity in the presence of 10% water (68.0 mPa·s^−1^). Therefore, increasing the temperature to 328.15 K and adding water up to 20% are very necessary and beneficial to obtain a higher solubility of carotenoids in phosphonium- and ammonium-based ILs.

Moreover, the phosphonium-based ILs presented a stronger ability than ammonium-based ILs to dissolve carotenoids. In particular, the ILs with Cl- showed a stronger solubility than the ILs with CF_3_COO^−^, and the ILs with longer alkyl chains facilitated the solubilization of carotenoids. Longer cationic alkyl side chains of the ILs resulted in stronger molecular vibrations, while a smaller ionic radius of anions resulted in more ions being transported and weaker van der Waals forces. Therefore, it could be observed that [P_4448_]Cl showed the highest solubility for carotenoids, while [N_4444_]CF_3_COO showed the lowest solubility. The solubilities of astaxanthin, β-carotene, and lutein in [P_4448_]Cl at 338.15 K and 20% water content were approximately 40 mg/100 g, 105 mg/100 g, and 5250 mg/100 g, respectively, which was 10 times that in [N_4444_]CF_3_COO. In addition, the solubility of different carotenoids in the ILs followed the trend lutein >> β-carotene- > astaxanthin. Lutein was highly polarized, and the solubility was hundreds of times higher than that reported in [43].

Methanol, ethanol, and acetone were the common organic solvents for natural dissolution and extraction [44]. The dissolution capacity of the methanol, ethanol, acetone were 0.02 mg/mL, 0.02 mg/mL, and 0.35 mg/mL at 35 °C, respectively [45]. Microemulsions had a large solubilization capacity for both hydrophilic and lipophilic compounds [46]. The solubilities of astaxanthin and lutein were 0.27 mg/mL and 12.50 mg/mL, respectively, in microemulsions composed of deep eutectic solvents (DL-menthol:acetic acid = 1:2), tween 80, and water [45]. In our study, the solubilities of astaxanthin and lutein in [P_4448_]Cl at 35 °C and 20% water content were 0.07 mg/mL and 15.07 mg/mL, respectively. These results indicate that [P_4448_]Cl aqueous solutions are good substitutes for organic solvents to improve the dissolution of carotenoids.

#### 2.3.2. Interaction Force of Carotenoids with ILs

**Figure 3:**
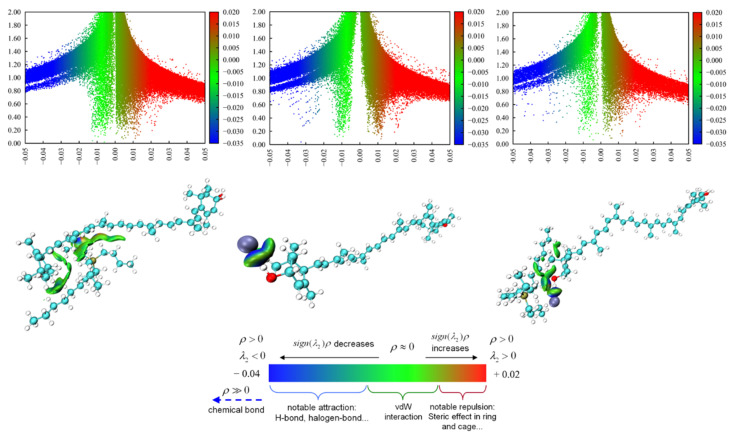
Isosurface diagrams, RDG (**top**) and independent gradient model, IGM (**bottom**) of [P_4448_]^+^-lutein (**left**), Cl^−1^–lutein (**middle**), and [P_4448_]Cl–lutein (**right**) calculated by molecular simulation.

DFT studies allow us to interpret the mechanism of ILs with carotenoids, bonding types, and the strength of the bonding in the considered IL–carotenoid systems. Figure S1 shows the optimized scatter diagrams of [P_4448_]^+^–carotenoid, Cl^−^–carotenoid, and [P_4448_]Cl^−^–carotenoid structures. To explore the interaction forces, the interactions between ions and carotenoids were investigated using the reduced density gradient (RDG) and independent gradient model (IGM). The RDG analysis can distinguish between regions of the system with different characteristics and clearly shows areas of strong attraction [47]. The interaction force for carotenoids with [P_4448_]Cl can be seen in Figure S2. For example, there is a large number of green scatter points perpendicular to the horizontal coordinate sign in the range of sign(*λ*_2_)*⍴*(*r*) of −0.01 to 0.01 au, indicating the presence of strong van der Waals interactions between conjugated double bonds in lutein with the cation of [P_4448_]Cl (Figure 3). Moreover, there are more dense blue scatters in the range of sign(*λ*_2_)*⍴*(*r*) of −0.03 to −0.05 au, suggesting hydrogen bonding between the H atom in lutein and the Cl^−^ atom in IL. 

The total interaction energy (*E_int_*) and energy gap (*ΔG*) for the optimized [P_4448_]Cl–carotenoid structures were calculated with the strength of [P_4448_]Cl–carotenoid interaction and are presented in Table 2. Large interaction energies were obtained for the studied IL–carotenoid clusters, which indicates a high affinity of IL parts resulting from either the anion or cation with the carotenoid structures. For astaxanthin, β-carotene, and lutein, [P_4448_]^+^ showed a larger *E_int_* than Cl^−1^, suggesting that the interactions are mostly localized around the cation sites rather than the anion sites. In the case of [P_4448_]Cl, the Eint value of IL–astaxanthin was the largest compared with others, whereas for astaxanthin, the solubility in [P_4448_]Cl was the lowest. In a previous study, the solubilities of astaxanthin and lutein in deep eutectic solvent (DES)-based microemulsions showed a good linear relationship with the interaction energy of DES–carotenoid [45]. Therefore, the strong solubility of carotenoids in [P_4448_]Cl were due to the van der Waals forces and hydrogen bonding that formed between them, and the IL cations play a major role in the interactions with the carotenoids. 

### 2.4. Carotenoid Stability in [P_4448_]Cl Aqueous Solution

Nowadays, natural pigments are becoming increasingly popular in the food and cosmetics industry. It was demonstrated that light, temperature, pH, and oxygen could affect the pigment stability in storage [48]. However, the effect of solvent nature on the pigment stability was always ignored in the previous literature. It was reported that 69% of astaxanthin was degraded after storage in hydroxypropyl-β-cyclodextrin at 4 °C for 120 h, and it was totally destroyed after storage at 50 °C for 32 h [49]. Storage of carotenoids at extreme pH values of <4 or >7 induces de-esterification and cis/trans isomerization of molecules [49]. In this study, the color stability and concentration change of carotenoids in [P_4448_]Cl aqueous solution at different temperatures and water contents was explored. Each [P_4448_]Cl aqueous solution containing astaxanthin, β-carotene, and lutein was stored in the dark for 12 days. Pictures of each sample stored from day 0 to day 12 are given in the Supplementary Materials (Figures S3–S7). Changes in ΔE, the concentration of carotenoids, and the relationship between ΔE and carotenoid concentration were measured to analyze the carotenoid stability in IL solutions.

**Figure 4:**
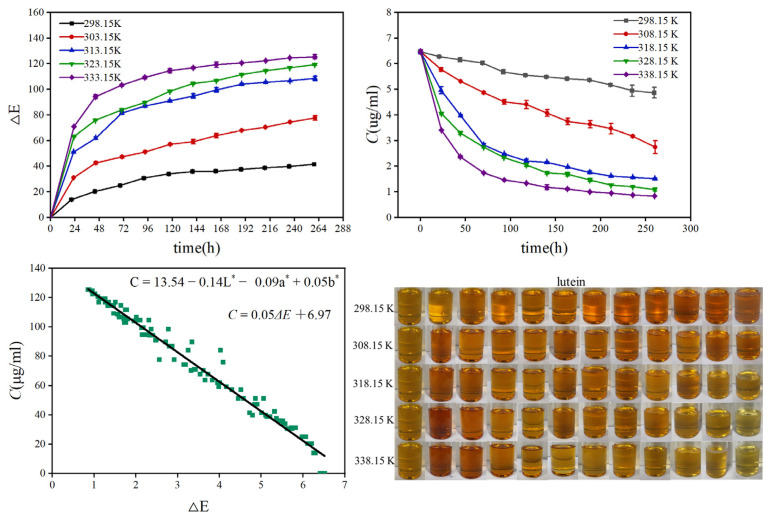
Changes in ΔE (total color deviation) and concentration (*C*) of lutein with time, the relationship between ΔE and lutein concentration, and pictures of the [P_4448_]Cl aqueous solution (*w_water_* = 20%) containing lutein during storage for 12 days at 298.15–338.15 K.

An example of lutein stability in [P_4448_]Cl aqueous solution (*w_water_* = 20%) at a temperature from 283.15 K to 338.15 K is shown in Figure 4.

#### 2.4.1. Color Stability

Figure S3 presents the appearance of a [P_4448_]Cl aqueous solutions containing astaxanthin, β-carotene, and lutein during storage. All the mixtures were clear and homogeneous when stored for 12 days. Most of the solutions (*w_water_* > 30%) containing astaxanthin were red at 298.15 K to 328.15 K, then changed to orange at 338.15 K. For β-carotene and lutein, the solutions were light orange, and slightly visible changes were perceivable by the human eye. 

The chromatic parameters (L*, a*, and b*) for all the solutions under different storage temperatures and water contents are shown in Figures S4 and S5, respectively. a* was the main parameter that reflects the color change of astaxanthin, and it decreased with increasing storage time. The value of b* is the main parameter that reflects the color change of β-carotene and lutein, and it also decreased with increasing storage time. ΔE was calculated using Equation (3) and is given in Figure S6. The larger the value of ΔE, the less stable the color of the sample. The ΔE value of all samples increased significantly at the beginning of storage, while it increased sightly with the extension of storage time, indicating that the carotenoid degraded continuously during storage. 

Moreover, the ΔE values of all [P_4448_]Cl aqueous solutions containing astaxanthin, β-carotene, and lutein followed a temperature-dependent trend, whereby the ΔE value was obviously higher at a high temperature (338.15 K) compared to a low temperature (298.15 K). By contrast, the value of ΔE for the samples containing 10% water was slightly lower than that containing 50% water at 298.15 K. The result indicated that carotenoids in [P_4448_]Cl aqueous solution have good stability at low temperature, and the effect of water content can be ignored. After 12 days of storage at 298.15 K, the ΔE value of [P_4448_]Cl solution containing astaxanthin, β-carotene, and lutein was approximately 20, 45, and 30, respectively. 

#### 2.4.2. Carotenoid Degradation 

The carotenoid concentration in all the solutions stored at different temperatures and water contents was also analyzed, and the data is shown in Figure S7. The degradation of astaxanthin, β-carotene, and lutein in all samples was related with the temperature and water content, whereby the degradation was more rapid at a high temperature (>318.15 K) or a high water content (>30%). After 12 days of storage at 298.15 K, the concentration of astaxanthin, β-carotene, and lutein in [P_4448_]Cl solution (*w_water_* = 20%) decreased by approximately 13.4%, 42.3%, and 15.3%, respectively. It is worth noting that the carotenoid degradation in [P_4448_]Cl solution was in agreement with the color change of the solution.

Moreover, the carotenoid degradation fitted a first-order kinetic reaction, expressed as follows [50]:

*ln* (*c/c*_0_) = *−kt* (1)

*t*_1/2_ = 0.693/*k* (2)

where *c* and *c*_0_ represent the carotenoids concentration at a specific storage time and at the initial time, respectively; *t* is the storage time (*h*); *t*_1/2_ is the half-life (*h*); and *k* is the rate constant (*h^−^*^1^).

High *R*^2^ (>95%) values for all [P_4448_]Cl solutions containing astaxanthin, β-carotene, and lutein were obtained, showing that the experimental data fit the first-order model. Similarly, the degradation and color change of astaxanthin in different atmosphere (air, vacuum) and storage temperatures (4, 15, 25 °C) and those of β-carotene in zein-carboxymethyl chitosan-tea polyphenol ternary composite nanoparticles stored in the dark at 5, 20, 37, and 60 °C for 7 days were found to follow a first-order kinetic reaction [51,52]. The kinetic parameters (*k*_1_ and *t*_1/2(h)_) obtained from the first-order model are listed in Table 3. Generally, the samples stored at low temperatures and water contents showed a low degradation rate. The *k*_1_ and *t*_1/2(h)_ values demonstrated that the stability of the carotenoids in the [P_4448_]Cl solution followed the trend lutein > astaxanthin > β-carotene.

#### 2.4.3. Correlation between Concentration of Carotenoids and Color Parameters

For all [P_4448_]Cl solutions containing carotenoids stored at different temperatures and water contents, varying degrees of color change and carotenoid degradation were observed. A previous study demonstrated a strong positive correlation between lutein color degradation and reduction in carotenoid content [53]. However, negative correlations were obtained for astaxanthin, astaxanthin ester, and lutein when stored in deep eutectic solvent-based microemulsions [45]. Hence, the relationship between the ΔE value and carotenoid content in the [P_4448_]Cl solutions was determined, and the results are shown in Table 3. It can be seen that the chromatic parameters (L*, a*, and b*) and ΔE are significantly related to carotenoid concentration. The equations were able to correlate the carotenoid concentrations throughout the chromatic parameters, as *R*^2^ values were around 0.87–0.98. The linear correlation demonstrate that the color parameters were an ideal index to conveniently monitor the carotenoid contents in IL solutions during storage.

## 3. Materials and Methods

### 3.1. Materials

**Figure 5:**
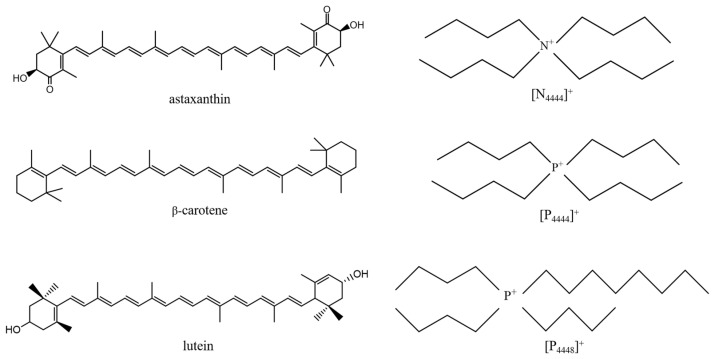
Chemical structures of astaxanthin, β-carotene, lutein, and the cations of ILs studied in this work.

Carotene standards (astaxanthin ≥ 98%, β-carotene ≥ 90%, and lutein ≥ 90%) were purchased from Aladdin Ltd. (Shanghai, China). Tetrabutylammonium bromide ([N_4444_]Br), tetrabutylammonium chloride ([N_4444_]Cl), tetrabutylammonium trifluoroacetate ([N_4444_]CF_3_COO), tetrabutylphosphonium bromide ([P_4444_]Br), tetrabutylphosphonium chloride ([P_4444_]Cl), tetrabutylphosphonium trifluoroacetate ([P_4444_]CF_3_COO), tributyloctylphosphonium bromide ([P_4448_]Br), tributyloctylphosphonium chloride ([P_4448_]Cl), and tributyloctylphosphonium trifluoroacetate ([P_4448_]CF_3_COO) were obtain from Cheng Jie Ltd. (Shanghai, China). The purity of all ILs was >99%. The chemical structures of the ILs, astaxanthin, β-carotene, and lutein are presented in Figure 5.

### 3.2. IL Properties

The physicochemical properties, including density, pH, conductivity, and viscosity of the IL/water mixtures (*w_water_* = 10% to 50%), were determined at atmospheric pressure and temperatures varying from *T* = (298.15 to 338.15 K) in steps of 10 K for the equal volume method. The samples were placed on a temperature-controlled chuck. The pH was determined using a pH meter (PHSJ-3F, Yidian Scientific Instrument Co., Ltd., Shanghai, China). The conductivity was determined using a conductivity meter (DDSJ-308A, Yidian Scientific Instrument Co., Ltd., Shanghai, China). The viscosity of the ILs was measured by a Malvern Kinexus Rheometer (Thermo, Waltham, MA, USA), and a parallel plate (diameter 20 mm) geometry was used with a fixed gap distance (1.0 mm) between the plates. 

### 3.3. Carotenoid Solubility

The equilibrium solubility method was based on the saturation shake-flask solubility technique [42]. When the temperature reached the set value (298.15 K to 338.15 K), the carotenoid standard was added into the mixtures of IL and water (*w_water_* = 10% to 50%) until it was in excess. An SHA-CA Water bath thermostat shaker (Changzhou Aohua Biotechnology Co., Ltd., Changzhou, China) was used to stir the mixture for more than 24 h until it was dissolved and balanced. After reaching the equilibrium point, the saturated solution was centrifuged and subsequently filtered through a syringe filter (0.45 μm). The solution was diluted with ethanol and analyzed using a UV–Vis spectrophotometer. The concentration of the diluted solution was determined from the calibration curve.

### 3.4. Molecular Simulation

The PM7 semi-empirical method in the MOPAC program was used to optimize the initial structures of ILs, astaxanthin, β-carotene, and lutein. The clustering was performed using an energy of 0.5 kcal/mol and a conformational difference of 0.25 Å. The configuration optimization was carried out at the B3LYP-D3/6-31G* basis set level by using density functional theory (DFT). The first three conformations that were finally optimized were subjected to a single-point energy calculation at the B3LYP-D3/Def2-TZVP.

### 3.5. Storage Stability of Carotenoids in IL Aqueous Solutions

#### 3.5.1. Color Changes

The color analysis samples were prepared by dissolving 1–2 mg of carotenoids into 20 mL of IL/water mixtures with ultrasound-assisted dissolution for 5 min. After filtering, the samples were incubated at different temperatures (298.15 K to 338.15 K) or different water contents (10% to 50%). To assessment of storage stability of different carotenoids in the IL/water mixtures, each sample was stored in the dark for 12 d. The L* (lightness), a*((+) red/(−) green), and b* ((+) yellow/(−) blue) color parameters were measured every 24 h using an automatism colorimeter (SC-80C, KangGuang Optical Instrument, CHN). The total color difference (Δ*E*) was calculated with the following equation:

(3) $$\Delta E^{2}={\left(L_{0}^{*}-L*\right)}^{2}+{\left(a_{0}^{*}-a*\right)}^{2}+{\left(b_{0}^{*}-b*\right)}^{2}$$

where $L_{0}^{*}$, $a_{0}^{*}$, and $b_{0}^{*}$ are the initial values of L*, a*, and b* color coordinates of the untreated samples, respectively. The color coordinates of samples that underwent storage or light treatment were L*, a*, and b*.

#### 3.5.2. Concentration Changes

A 1–2 mg sample of carotenoids was dissolved into 20 mL of IL aqueous solution with ultrasound-assisted dissolution for 5  min. As storage time increased, the concentrations of astaxanthin, β-carotene, and lutein in the IL/water mixtures were determined by measuring the absorbance using a microplate reader (Synergy H1, USA). The carotenoid standards were dissolved in an anhydrous ethanol solution, and the standards were diluted with ethanol to different concentrations until the absorbance was between 0.2 and 1.0 absorbance units. After plotting the standard curve, the carotenoid concentrations could be derived from the absorbance. 

### 3.6. Statistical Analysis 

All experiments were performed in triplicate and the data were expressed as the mean ± standard deviation (SD). One-way analysis of variance (ANOVA) (Tukey’s procedure at a confidence level of 95%) was applied, and significant differences were compared by Tukey’s test at *p* < 0.05 using SPSS version 26.0.

## 4. Conclusions

Developing a new extraction solvent for carotenoids and studying their storage stability in solvents is beneficial for their application. In this study, ammonium- and phosphonium-based ILs were used as alternatives to organic solvents for astaxanthin, β-carotene, and lutein. The effects of the solvent nature, temperature, and water content on the physicochemical properties, carotenoid solubility, and storage stability were evaluated. The viscosity of all studied ILs was lower than 100 mPa·s^−1^ at 298.15–338.15 K at a water content of 20%. [P_4448_]Cl presented the highest dissolving capacity at 338.15 K with a water content of 10%, which was higher than the organic solvent and imidazolium IL. The strong solubility of carotenoids in [P_4448_]Cl was due to the interactions of IL cations with the carotenoids. Moreover, the solubility and stability of the carotenoids in [P_4448_]Cl solution followed the trends lutein >> β-carotene > astaxanthin, and lutein > astaxanthin > β-carotene, respectively. The carotenoid degradation followed a first-order kinetic reaction, and the chromatic parameters (L*, a* and b*) and Δ*E* are significantly related to carotenoid concentration. Therefore, the color parameters were demonstrated as an ideal index to conveniently monitor the carotenoid contents in IL solutions during storage. This study provides a valuable reference for the extraction of carotenoids using ammonium- and phosphonium-based ILs, as well as the application of the extraction solution in the pharmaceutical, cosmeceutical, food, and feed industries.

## Figures and Tables

**Table 1:** Phase behavior of IL–water mixtures at 298.15 K.

IL	Water Content				
	*w_water_* = 10%	*w_water_* = 20%	*w_water_* = 30%	*w_water_* = 40%	*w_water_* = 50%
[N_4444_]Br	○	○	○	○	○
[N_4444_]Cl	○	○	○	○	○
[N_4444_]CF_3_COO	○	○	○	○	○
[P_4444_]Br	○	○	○	○	○
[P_4444_]Cl	○	○	○	○	○
[P_4444_]CF_3_COO	○	○	○	○	○
[P_4448_]Br	○	×	×	×	×
[P_4448_]Cl	○	○	○	○	○
[P_4448_]CF_3_COO	×	×	×	×	×

“○” indicates a miscible homogeneous phase, “×” indicates stratification without miscibility.

**Table 2:** Interaction energies (*E*_int_) between [P_4448_]^+^, Cl^−1^, and [P_4448_]Cl with carotenoids. *E_complex_* represents the energy of the complex. *E_complex(Carotenoids)_* and *E_complex (ILs)_* represent the energy of carotenoids (astaxanthin, β-carotene, and lutein) molecules and ionic liquids ([P_4448_]^+^, Cl^−1^, and [P_4448_]Cl) molecules, respectively.

System	*E_complex_* (×10^3^ kcal/mol)	*E_complex(Carotenoids)_* (×10^3^ kcal/mol)	*E_complex (ILs)_* (×10^3^ kcal/mol)	*E_int_* (kcal/mol)
[P_4448_]^+^–astaxanthin	−2314.6202	−1605.7453	−708.8368	−38.07
[P_4448_]^+^–β-carotene	−1686.2509	−977.3796	−708.8472	−24.18
[P_4448_]^+^–lutein	−1779.3847	−1071.0201	−708.3377	−26.96
Cl^−1^–astaxanthin	−1894.5762	−1605.7416	−288.7999	−34.69
Cl^−1^–β-carotene	−1266.1982	−977.3804	−288.7960	−21.78
Cl^−1^–lutein	−1359.6329	−1071.0182	−288.5885	−26.24
[P_4448_]Cl–astaxanthin	−2603.4850	−1605.7341	−997.7130	−37.86
[P_4448_]Cl–β-carotene	−1973.7142	−976.6761	−997.0171	−21.05
[P_4448_]Cl–lutein	−2068.0591	−1071.0202	−997.01487	−24.10

**Table 3:** The rate constants (*k*_1_, h^−1^), *t*_1/2(h)_ and the relationship between the concentration of carotenoids and chromatic parameters.

Carotenoid	*w_water_*	*T* (K)	*k*_1_ (h^−1^)	*t*_1/2_ (h)	*f* (*C*) = *f* (L*, a*, b*) = *f* (Δ*E*)	*R* ^2^
Astaxanthin	10	298.15	−0.0028	247.50	*C_As_*_t_ = −28.14 + 0.28L* + 0.49a* − 0.04b*	0.9050
	20	298.15	−0.0026	266.54	*C_Ast_* = 0.18Δ*E* +14.00	0.8727
	30	298.15	−0.0024	288.75		
	40	298.15	−0.0032	216.56		
	50	298.15	−0.0056	123.75		
	20	308.15	−0.0029	238.97		
	20	318.15	−0.0036	192.50		
	20	328.15	−0.0050	138.60		
	20	338.15	−0.0103	67.28		
β-Carotene	10	298.15	−0.0016	433.13	*C_Car_* = 6.06 + 0.06L* + 0.04a* + 0.10b*	0.9823
	20	298.15	−0.0027	256.67	*C_Car_* = −0.11Δ*E* + 7.92	0.9545
	30	298.15	−0.0029	238.97		
	40	298.15	−0.0037	187.30		
	50	298.15	−0.0054	128.33		
	20	308.15	−0.0038	182.37		
	20	318.15	−0.0050	138.60		
	20	328.15	−0.0065	106.62		
	20	338.15	−0.0089	77.87		
Lutein	10	298.15	−0.0011	630.00	*C_Lut_* = 13.54 − 0.14L* − 0.09a* + 0.05b*	0.8906
	20	298.15	−0.0012	577.50	*C_Lut_* = −0.05Δ*E* + 6.97	0.9607
	30	298.15	−0.0015	462.00		
	40	298.15	−0.0022	315.00		
	50	298.15	−0.0033	210.00		
	20	308.15	−0.0033	210.00		
	20	318.15	−0.0068	101.91		
	20	328.15	−0.0080	86.63		
	20	338.15	−0.0099	70.00		

## Data Availability

The data presented in this study are available in insert article and Supplementary Material here.

## Supplementary Materials

The following supporting information can be downloaded at: https://www.mdpi.com/article/10.3390/molecules28083618/s1. Table S1: Effect of temperature on the densities (*ρ*), molar volumes (*Vm*), isobaric expansion coefficient (*α_p_*) pH, conductivity (*σ*), and viscosity(*η*) of the ILs containing 20% water at atmospheric pressure. Table S2: Effect of water content on the densities (*ρ*), molar volumes (*V*_m_), *pH*, conductivity (*σ*), and viscosity(*η*) of the IL/water systems at T = (298.15) K and atmospheric pressure. Figure S1: The scatter diagrams of [P_4448_]^+^–astaxanthin (A), Cl^−^–astaxanthin (B), [P_4448_]Cl–astaxanthin (C), [P_4448_]^+^–β-carotene (D), Cl^−^–β-carotene (E), [P_4448_]Cl–β-carotene (F), [P_4448_]^+^–lutein (G), Cl^−^–lutein (H), and [P_4448_]Cl–lutein (I). Figure S2: Plots of reduced density gradient (RDG) versus electron density multiplied by the sign of λ_2_: [P_4448_]^+^–astaxanthin (A), Cl^−^–astaxanthin (B), [P_4448_]Cl–astaxanthin (C), [P_4448_]^+^–β-carotene (D), Cl^−^–β-carotene (E), [P_4448_]Cl–β-carotene (F), [P_4448_]^+^–lutein (G), Cl^−^–lutein (H), and [P_4448_]Cl–lutein (I). Figure S3: Color parameters (L*, a*, and b*) of [P_4448_]Cl aqueous solution containing astaxanthin (top), β-carotene (middle), and lutein (bottom) as a function of water content stored in the dark at 25 °C. Figure S4: Color parameters (L*, a*, and b*) of [P_4448_]Cl aqueous solution containing astaxanthin (top), β-carotene (middle), and lutein (bottom) as a function of temperature stored at 20% water in the dark. Figure S5: Pictures of [P_4448_]Cl aqueous solution containing astaxanthin (top), β-carotene (middle), and lutein (bottom) as a function of different storage conditions from 0 day to 12 day. C_0_(μg/mL) represents the initial concentration of carotenoids in the solution. Figure S6: ΔE of [P_4448_]Cl aqueous solutions containing astaxanthin (top), β-carotene (middle), and lutein (bottom) for 12 days of storage. C_0_(μg/mL) represents the initial concentration of carotenoids in the solution. Figure S7: Concentration(C) of astaxanthin (top), β-carotene (middle), lutein (bottom) in [P_4448_]Cl aqueous solution for 12 days of storage.

## References

[1] Yu J., Liu X., Zhang L., Shao P., Wu W., Chen Z., Li J., Renard C.M.G.C. (2022). An overview of carotenoid extractions using green solvents assisted by Z-isomerization. Trends Food Sci. Technol..

[2] Wen Lee H., Bi X., Jeyakumar Henry C. (2022). Carotenoids, tocopherols and phylloquinone content of 26 green leafy vegetables commonly consumed in Southeast Asia. Food Chem..

[3] Luana Carvalho de Queiroz J., Medeiros I., Costa Trajano A., Piuvezam G., Clara de Franca Nunes A., Souza Passos T., Heloneida de Araujo Morais A. (2022). Encapsulation techniques perfect the antioxidant action of carotenoids: A systematic review of how this effect is promoted. Food Chem..

[4] Hajizadeh-Sharafabad F., Ghoreishi Z., Maleki V., Tarighat-Esfanjani A. (2019). Mechanistic insights into the effect of lutein on atherosclerosis, vascular dysfunction, and related risk factors: A systematic review of in vivo, ex vivo and in vitro studies. Pharmacol. Res..

[5] Madaan T., Choudhary A.N., Gyenwalee S., Thomas S., Mishra H., Tariq M., Vohora D., Talegaonkar S. (2017). Lutein, a versatile phyto-nutraceutical: An insight on pharmacology, therapeutic indications, challenges and recent advances in drug delivery. PharmaNutrition.

[6] Zhuang D., He N., Khoo K.S., Ng E.P., Chew K.W., Ling T.C. (2022). Application progress of bioactive compounds in microalgae on pharmaceutical and cosmetics. Chemosphere.

[7] Tiwari S., Upadhyay N., Singh A.K. (2022). Stability assessment of emulsion of carotenoids extracted from carrot bio-waste in flaxseed oil and its application in food model system. Food Biosci..

[8] Zhang Z., Chen W., Zhou X., Deng Q., Dong X., Yang C., Huang F. (2021). Astaxanthin-loaded emulsion gels stabilized by Maillard reaction products of whey protein and flaxseed gum: Physicochemical characterization and in vitro digestibility. Food Res. Int..

[9] Lu Q., Li H., Zou Y., Liu H., Yang L. (2021). Astaxanthin as a microalgal metabolite for aquaculture: A review on the synthetic mechanisms, production techniques, and practical application. Algal Res..

[10] Díaz-Gómez J., Moreno J.A., Angulo E., Sandmann G., Zhu C., Ramos A.J., Capell T., Christou P., Nogareda C. (2017). High-carotenoid biofortified maize is an alternative to color additives in poultry feed. Anim. Feed Sci. Technol..

[11] Ren Y., Deng J., Huang J., Wu Z., Yi L., Bi Y., Chen F. (2021). Using green alga *Haematococcus pluvialis* for astaxanthin and lipid co-production: Advances and outlook. Bioresour. Technol..

[12] Mussagy C.U., Farias F.O., Bila N.M., Giannini M.J.S.M., Pereira J.F.B., Santos-Ebinuma V.C., Pessoa A. (2022). Recovery of β-carotene and astaxanthin from *Phaffia rhodozyma* biomass using aqueous solutions of cholinium-based ionic liquids. Sep. Purif. Technol..

[13] Britton G. (2020). Carotenoid research: History and new perspectives for chemistry in biological systems. Biochim. Biophys. Acta Mol. Cell Biol. Lipids.

[14] Dong S., Huang Y., Zhang R., Wang S., Liu Y. (2014). Four different methods comparison for extraction of astaxanthin from green alga *Haematococcus pluvialis*. Sci. World J..

[15] Gea-Botella S., Agullo L., Marti N., Martinez-Madrid M.C., Lizama V., Martin-Bermudo F., Berna G., Saura D., Valero M. (2021). Carotenoids from persimmon juice processing. Food Res. Int..

[16] Mero A., Guglielmero L., D’Andrea F., Pomelli C.S., Guazzelli L., Koutsoumpos S., Tsonos G., Stavrakas I., Moutzouris K., Mezzetta A. (2022). Influence of the cation partner on levulinate ionic liquids properties. J. Mol. Liq..

[17] Poole C.F., Atapattu S.N. (2021). Determination of physicochemical properties of ionic liquids by gas chromatography. J. Chromatogr. A.

[18] Kaur G., Kumar H., Singla M. (2022). Diverse applications of ionic liquids: A comprehensive review. J. Mol. Liq..

[19] Shen Q., Zhu T., Wu C., Xu Y., Li C. (2022). Ultrasonic-assisted extraction of zeaxanthin from *Lycium barbarum* L. with composite solvent containing ionic liquid: Experimental and theoretical research. J. Mol. Liq..

[20] Zhu Y., Li X., Wang Y., Ren L., Zhao Q. (2021). Lutein extraction by imidazolium-based ionic liquid-water mixture from dried and fresh Chlorella sp. Algal Res..

[21] Li J., Wang Z., Yao S., Song H. (2020). Aqueous solubilization and extraction of curcumin enhanced by imidazolium, quaternary ammonium, and tropine ionic liquids, and insight of ionic liquids-curcumin interaction. J. Mol. Liq..

[22] Bi W., Tian M., Row K.H. (2013). Evaluation of alcohol-based deep eutectic solvent in extraction and determination of flavonoids with response surface methodology optimization. J. Chromatogr. A.

[23] Wang X., Chi Y., Mu T. (2014). A review on the transport properties of ionic liquids. J. Mol. Liq..

[24] Gao J., Guo J., Nie F., Ji H., Liu S. (2017). LCST-Type Phase Behavior of Aqueous Biphasic Systems Composed of Phosphonium-Based Ionic Liquids and Potassium Phosphate. J. Chem. Eng. Data.

[25] Kono S., Kazama H., Mori T., Arai T., Takao K. (2018). Significant Acceleration of PGMs Extraction with UCST-Type Thermomorphic Ionic Liquid at Elevated Temperature. ACS Sustain. Chem. Eng..

[26] Boli E., Katsavrias T., Voutsas E. (2020). Viscosities of pure protic ionic liquids and their binary and ternary mixtures with water and ethanol. Fluid Phase Equilibria.

[27] Bobrova L.S., Danilov F.I., Protsenko V.S. (2016). Effects of temperature and water content on physicochemical properties of ionic liquids containing CrCl_3_·xH_2_O and choline chloride. J. Mol. Liq..

[28] Da-Yong S., Jing C. (2014). Hydrogen-Bonding Interactions between Ionic Liquid 1-Ethyl-3-methylimidazolium Trifluoromethanesulfonate and Water. Acta Phys.-Chim. Sin..

[29] Kartikawati N.A., Safdar R., Lal B., Mutalib M.I.B.A., Shariff A.M. (2018). Measurement and correlation of the physical properties of aqueous solutions of ammonium based ionic liquids. J. Mol. Liq..

[30] Sharma S., Sharma S., Singh J., Singh M., Sharma A.K., Sharma M. (2022). Study on molecular interactions of l-leucine in aqueous ionic liquid (1-butyl-3-methylimidazolium tetrafluoroborate) [C4mim][BF4] solution using density, speed of sound and viscosity measurements at various temperatures. J. Chem. Thermodyn..

[31] Wang R., Wang Y., Guo W., Zeng M. (2021). Stability and bioactivity of carotenoids from *Synechococcus* sp. PCC 7002 in Zein/NaCas/Gum Arabic composite nanoparticles fabricated by pH adjustment and heat treatment antisolvent precipitation. Food Hydrocoll..

[32] Nayana Lakshmi S., Bahadur P., Dutta Choudhury S. (2021). Photoinduced electron transfer reactions in mixed micelles of a star block copolymer and surface active ionic liquids: Role of the anion. J. Mol. Liq..

[33] van Osch D.J.G.P., Dietz C.H.J.T., van Spronsen J., Kroon M.C., Gallucci F., van Sint Annaland M., Tuinier R. (2019). A Search for Natural Hydrophobic Deep Eutectic Solvents Based on Natural Components. ACS Sustain. Chem. Eng..

[34] Chávez-Castellanos Á.E., Aguilar-Martinez M., Reyna-González J.M. (2022). Effect of water and ions on the rheological behavior of a low viscosity ammonium-based ionic liquid. Fluid Phase Equilibria.

[35] Królikowska M., Lipiński P., Maik D. (2014). Density, viscosity and phase equilibria study of {ethylsulfate-based ionic liquid+water} binary systems as a function of temperature and composition. Thermochim. Acta.

[36] Zhao Y., Tian L., Pei Y., Wang H., Wang J. (2018). Effect of Anionic Structure on the LCST Phase Behavior of Phosphonium Ionic Liquids in Water. Ind. Eng. Chem. Res..

[37] Gao J., Fang C., Lin Y., Nie F., Ji H., Liu S. (2020). Enhanced extraction of astaxanthin using aqueous biphasic systems composed of ionic liquids and potassiumphosphate. Food Chem..

[38] Wang J., Li Y., Liu H., Tong J. (2022). Surface tension, viscosity and electrical conductivity characteristics of new ether-functionalized ionic liquids. J. Mol. Liq..

[39] Myrdek T., Popescu C., Kunz W. (2021). Physical-chemical properties of newly synthesized tetraalkylammonium alkyl ether carboxylate ionic liquids. J. Mol. Liq..

[40] Yildirim A., Szymoniak P., Sentker K., Butschies M., Buhlmeyer A., Huber P., Laschat S., Schonhals A. (2018). Dynamics and ionic conductivity of ionic liquid crystals forming a hexagonal columnar mesophase. Phys. Chem. Chem. Phys..

[41] Ge M., Fang T., Zhou G., Li C., Li Y., Liu X. (2022). Insight into the dual effect of water on lignin dissolution in ionic liquids. Int. J. Biol. Macromol..

[42] Mirheydari S.N., Barzegar-Jalali M., Shekaari H., Martinez F., Jouyban A. (2019). Experimental determination and correlation of lamotrigine solubility in aqueous mixtures of 1-octyl-3-methylimidazolium bromide ionic liquid at various temperatures. J. Chem. Thermodyn..

[43] Murador D.C., De Souza Mesquita L.M., Neves B.V., Braga A.R.C., Martins P.L.G., Zepka L.Q., De Rosso V.V. (2021). Bioaccessibility and cellular uptake by Caco-2 cells of carotenoids and chlorophylls from orange peels: A comparison between conventional and ionic liquid mediated extractions. Food Chem..

[44] Mercadante A.Z., Rodrigues D.B., Petry F.C., Mariutti L.R.B. (2017). Carotenoid esters in foods—A review and practical directions on analysis and occurrence. Food Res. Int..

[45] Li Y., Hu K., Huang C., Hu Y., Ji H., Liu S., Gao J. (2022). Improvement of solubility, stability and antioxidant activity of carotenoids using deep eutectic solvent-based microemulsions. Colloids Surf. B Biointerfaces.

[46] Shao M., Chen M., Fan M., Luo G., Jin C., Huang Z. (2021). Microemulsion system constructed with a new cyano-functionalized ionic liquid for the extraction of Pd(II) and preparation of palladium nanoparticles. Sep. Purif. Technol..

[47] Liu X., Fang J., Zheng W., Tan Z., Zheng X., Di J. (2021). Study on desulfurization mechanism of ionic liquid extractant based on Gaussian quantitative calculation. Comput. Theor. Chem..

[48] Nowacka M., Dadan M., Janowicz M., Wiktor A., Witrowa-Rajchert D., Mandal R., Pratap-Singh A., Janiszewska-Turak E. (2021). Effect of nonthermal treatments on selected natural food pigments and color changes in plant material. Compr. Rev. Food Sci. Food Saf..

[49] Martinez-Delgado A.A., Khandual S., Villanueva-Rodriguez S.J. (2017). Chemical stability of astaxanthin integrated into a food matrix: Effects of food processing and methods for preservation. Food Chem..

[50] Huang L., Li D., Ma Y., Liu Y., Liu G., Wang Y., Tan B. (2022). Dietary fatty acid-mediated protein encapsulation simultaneously improving the water-solubility, storage stability, and oral absorption of astaxanthin. Food Hydrocoll..

[51] Niamnuy C., Devahastin S., Soponronnarit S., Vijaya Raghavan G.S. (2008). Kinetics of astaxanthin degradation and color changes of dried shrimp during storage. J. Food Eng..

[52] Ba C., Fu Y., Niu F., Wang M., Jin B., Li Z., Chen G., Zhang H., Li X. (2020). Effects of environmental stresses on physiochemical stability of beta-carotene in zein-carboxymethyl chitosan-tea polyphenols ternary delivery system. Food Chem..

[53] Qian C., Decker E.A., Xiao H., McClements D.J. (2012). Physical and chemical stability of beta-carotene-enriched nanoemulsions: Influence of pH, ionic strength, temperature, and emulsifier type. Food Chem..

