# Supplementary material for: Solubility and Stability of Carotenoids in Ammonium- and Phosphonium-Based Ionic Liquids: Effect of Solvent Nature, Temperature and Water
Source: Molecules. 2023 Apr 21;28(8):3618. doi: 10.3390/molecules28083618 (PMC10143741; doi:10.3390/molecules28083618)
Supplement: Supplementary file 1 [file molecules-28-03618-s001.zip › molecules-2349108-supplementary.pdf]

# Supplementary materials

**Table S1** Effect of temperature on the densities ($\rho$), molar volumes ($V_m$), Isobaric expansion coefficient ($\alpha_p$) pH, conductivity ($\sigma$) and viscosity($\eta$) of the ILs containing 20% water at atmospheric pressure

*The ND means not measurable

| IL | Temperature (K) | $\rho$ (g/cm$^3$) | $V_m$ (cm$^3$·mol$^{-1}$) | $\alpha_p$ | pH | $\sigma$ (ms·cm$^{-1}$) | $\eta$ (mpa·s) |
|---|---|---|---|---|---|---|---|
| $[N_{4444}]Br$ | 298.15 | 1.0483 | 57.7415 | 4.7068 | 6.66 | 5.23 | 58.90 |
| | 308.15 | 1.0432 | 58.0221 | 4.7290 | 6.65 | 7.08 | 41.92 |
| | 318.15 | 1.0386 | 58.2796 | 4.7515 | 6.62 | 8.69 | 32.11 |
| | 328.15 | 1.0322 | 58.6415 | 4.7742 | 6.50 | 10.31 | 24.97 |
| | 338.15 | 1.0265 | 58.9660 | 4.7971 | 6.40 | 11.71 | 20.82 |
| $[N_{4444}]Cl$ | 298.15 | 0.9410 | 62.5307 | 5.2865 | 8.19 | 6.77 | 55.12 |
| | 308.15 | 0.9319 | 63.1440 | 5.3146 | 7.78 | 8.35 | 40.83 |
| | 318.15 | 0.9294 | 63.3132 | 5.3430 | 7.91 | 10.53 | 27.63 |
| | 328.15 | 0.9240 | 63.6832 | 5.3717 | 7.72 | 12.12 | 21.89 |
| | 338.15 | 0.9189 | 64.0360 | 5.4007 | 7.84 | ND | 18.25 |
| $[N_{4444}]CF_3COO$ | 298.15 | 1.0280 | 59.8992 | 5.7859 | 10.97 | 4.15 | 38.33 |
| | 308.15 | 1.0190 | 60.4295 | 5.8196 | 10.77 | 5.22 | 29.20 |
| | 318.15 | 1.0131 | 60.7796 | 5.8537 | 10.79 | 6.63 | 23.16 |
| | 328.15 | 1.0051 | 61.2652 | 5.8881 | 11.03 | 7.86 | 17.45 |
| | 338.15 | 0.9987 | 61.6566 | 5.9230 | 11.13 | 8.50 | 14.69 |
| $[P_{4444}]Br$ | 298.15 | 1.0353 | 59.5703 | 6.0967 | 2.52 | 7.92 | 46.23 |
| | 308.15 | 1.0256 | 60.1387 | 6.1103 | 2.42 | 10.15 | 29.61 |
| | 318.15 | 1.0179 | 60.6012 | 6.1525 | 2.09 | 12.02 | 26.10 |
| | 328.15 | 1.0110 | 61.0184 | 6.2149 | 1.92 | 12.88 | 22.74 |
| | 338.15 | 1.0046 | 61.4121 | 6.2565 | 1.71 | ND | 20.78 |
| $[P_{4444}]Cl$ | 298.15 | 0.9475 | 62.8605 | 6.4350 | 6.32 | 7.44 | 43.08 |
| | 308.15 | 0.9388 | 63.4383 | 6.4767 | 6.21 | 9.37 | 30.49 |
| | 318.15 | 0.9373 | 63.5418 | 6.5189 | 6.17 | 11.59 | 24.63 |
| | 328.15 | 0.9289 | 64.1151 | 6.5617 | 6.08 | 12.60 | 19.59 |
| | 338.15 | 0.9248 | 64.4014 | 6.6050 | 5.94 | ND | 16.19 |
| $[P_{4444}]CF_3COO$ | 298.15 | 1.0293 | 60.7481 | 6.9040 | 4.61 | 5.35 | 30.31 |
| | 308.15 | 1.0251 | 61.2432 | 6.9589 | 4.47 | 7.02 | 23.85 |
| | 318.15 | 1.0219 | 61.7074 | 7.0147 | 4.43 | 8.58 | 19.27 |
| | 328.15 | 1.0145 | 62.1128 | 7.0714 | 4.23 | 9.59 | 15.52 |
| | 338.15 | 1.0112 | 62.6953 | 7.1290 | 4.23 | 10.89 | 13.84 |
| $[P_{4448}]Cl$ | 298.15 | 0.9369 | 65.3378 | 7.4691 | 1.71 | 4.49 | 66.32 |
| | 308.15 | 0.9261 | 66.0998 | 7.5172 | 1.69 | 5.37 | 47.59 |
| | 318.15 | 0.9208 | 66.4838 | 7.5659 | 1.64 | 6.59 | 35.11 |
| | 328.15 | 0.9164 | 66.7994 | 7.6153 | 1.59 | 7.80 | 26.44 |
| | 338.15 | 0.9113 | 67.1733 | 7.6654 | 1.57 | 8.82 | 21.34 |

**Table S2** Effect of water content on the densities ($\rho$), molar volumes ($V_m$), *pH,* conductivity ($\sigma$) and viscosity($\eta$) of the IL/ water systems at T = (298.15) K and at atmospheric pressure.

| IL | $w_{water}$ | $\rho$ (g/cm$^3$) | $V_m$(cm$^3$·mol$^{-1}$) | pH | $\sigma$ (ms·cm$^{-1}$) | $\eta$ (mpa·s) |
|---|---|---|---|---|---|---|
| $[N_{4444}]Br$ | 10% | 1.0504 | 105.4439 | 6.59 | 1.93 | 118.90 |
| | 20% | 1.0483 | 66.2872 | 6.23 | 6.47 | 58.90 |
| | 30% | 1.0437 | 48.3886 | 6.09 | 11.03 | 25.66 |
| | 40% | 1.0368 | 38.2450 | 6.01 | 14.66 | 18.36 |
| | 50% | 1.0324 | 31.5753 | 5.89 | 16.86 | 12.49 |
| $[N_{4444}]Cl$ | 10% | 0.9570 | 112.5789 | 7.52 | 2.43 | 106.90 |
| | 20% | 0.9642 | 71.9081 | 7.40 | 7.31 | 55.17 |
| | 30% | 0.9686 | 52.3921 | 7.33 | 13.09 | 25.30 |
| | 40% | 0.9722 | 41.5489 | 7.24 | 17.09 | 18.05 |
| | 50% | 0.9779 | 34.2618 | 7.04 | ND | 10.74 |
| $[N_{4444}]CF_3COO$ | 10% | 1.0320 | 109.6201 | 11.01 | 1.43 | 60.10 |
| | 20% | 1.0290 | 68.1774 | 10.55 | 4.10 | 38.33 |
| | 30% | 1.0281 | 49.5023 | 10.10 | 5.69 | 26.33 |
| | 40% | 1.0249 | 39.0719 | 9.72 | 6.81 | 16.85 |
| | 50% | 1.0212 | 32.2960 | 9.38 | 7.76 | 11.41 |
| $[P_{4444}]Br$ | 10% | 1.0469 | 107.7884 | 6.26 | 2.29 | 81.76 |
| | 20% | 1.0424 | 67.3280 | 5.59 | 7.44 | 46.23 |
| | 30% | 1.0392 | 49.2676 | 5.14 | 12.26 | 21.82 |
| | 40% | 1.0335 | 39.1777 | 4.81 | 17.16 | 16.13 |
| | 50% | 1.0285 | 32.4669 | 4.63 | ND | 12.00 |
| $[P_{4444}]Cl$ | 10% | 0.9552 | 115.8498 | 6.25 | 2.37 | 94.61 |
| | 20% | 0.9651 | 72.9427 | 5.71 | 6.96 | 43.08 |
| | 30% | 0.9734 | 53.0244 | 5.31 | 11.63 | 25.62 |
| | 40% | 0.9797 | 41.5886 | 5.23 | 14.94 | 17.04 |
| | 50% | 0.9817 | 34.2838 | 5.19 | 16.5 | 12.29 |
| $[P_{4444}]CF_3COO$ | 10% | 1.03025 | 115.6351 | 4.94 | 1.39 | 52.10 |
| | 20% | 1.02505 | 70.9226 | 4.61 | 4.52 | 30.31 |
| | 30% | 1.02185 | 51.1910 | 4.34 | 5.92 | 20.33 |
| | 40% | 1.0145 | 40.4593 | 4.05 | 7.02 | 16.30 |
| | 50% | 1.0112 | 33.1357 | 3.84 | 7.93 | 12.41 |
| $[P_{4448}]Cl$ | 10% | 0.94385 | 124.7643 | 1.96 | 1.83 | 124.50 |
| | 20% | 0.95185 | 76.3259 | 1.71 | 5.66 | 66.32 |
| | 30% | 0.95785 | 55.0911 | 1.62 | 8.25 | 38.33 |
| | 40% | 0.9605 | 43.0576 | 1.63 | 9.99 | 29.97 |
| | 50% | 0.9669 | 35.1671 | 1.69 | 11.63 | 17.88 |

*The ND means not measurable

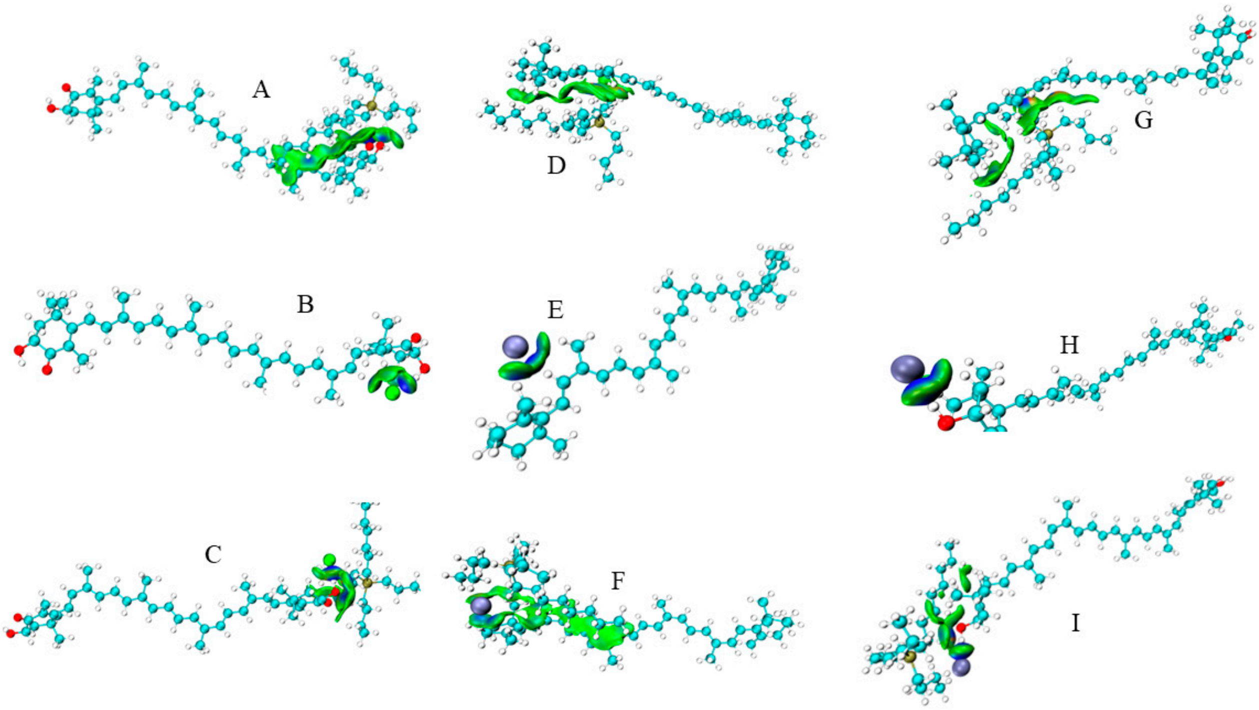


**Figure S1** The scatter diagrams of $[P_{4448}]^+$-astaxanthin (A), $Cl^-$-astaxanthin (B), $[P_{4448}]Cl$- astaxanthin (C), $[P_{4448}]^+$-β-carotene (D), $Cl^-$-β-carotene (E), $[P_{4448}]Cl$-β-carotene (F), $[P_{4448}]^+$-lutein (G), $Cl^-$-lutein (H), and $[P_{4448}]Cl$-lutein (I)

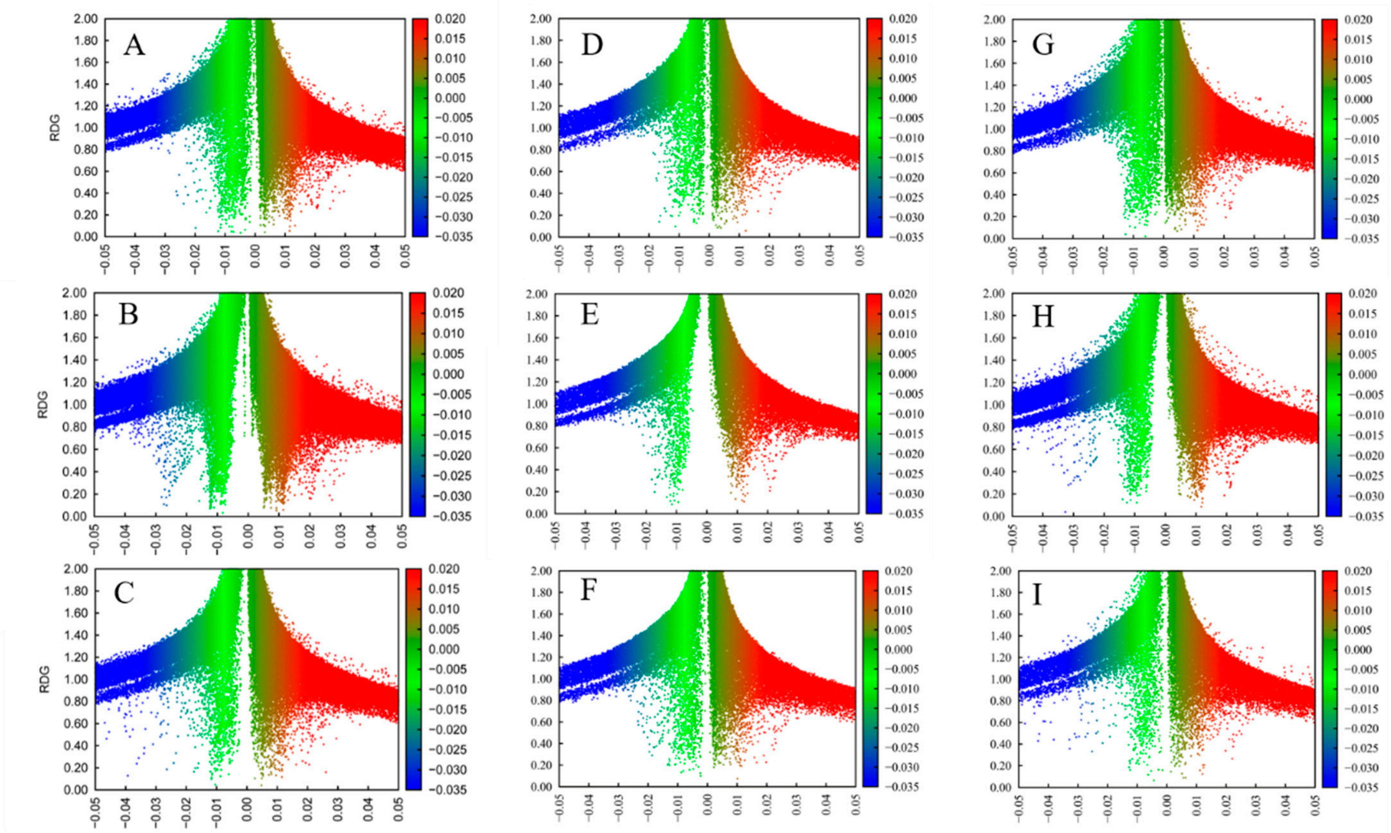


**Figure S2** Plots of reduced density gradient (RDG) versus electron density multiplied by the sign of $\lambda_2$: $[P_{4448}]^+$-astaxanthin (A), $Cl^-$-astaxanthin (B), $[P_{4448}]Cl$-astaxanthin (C), $[P_{4448}]^+$-β-carotene (D), $Cl^-$-β-carotene (E), $[P_{4448}]Cl$-β-carotene (F), $[P_{4448}]^+$-lutein (G), $Cl^-$-lutein (H), and $[P_{4448}]Cl$-lutein (I).

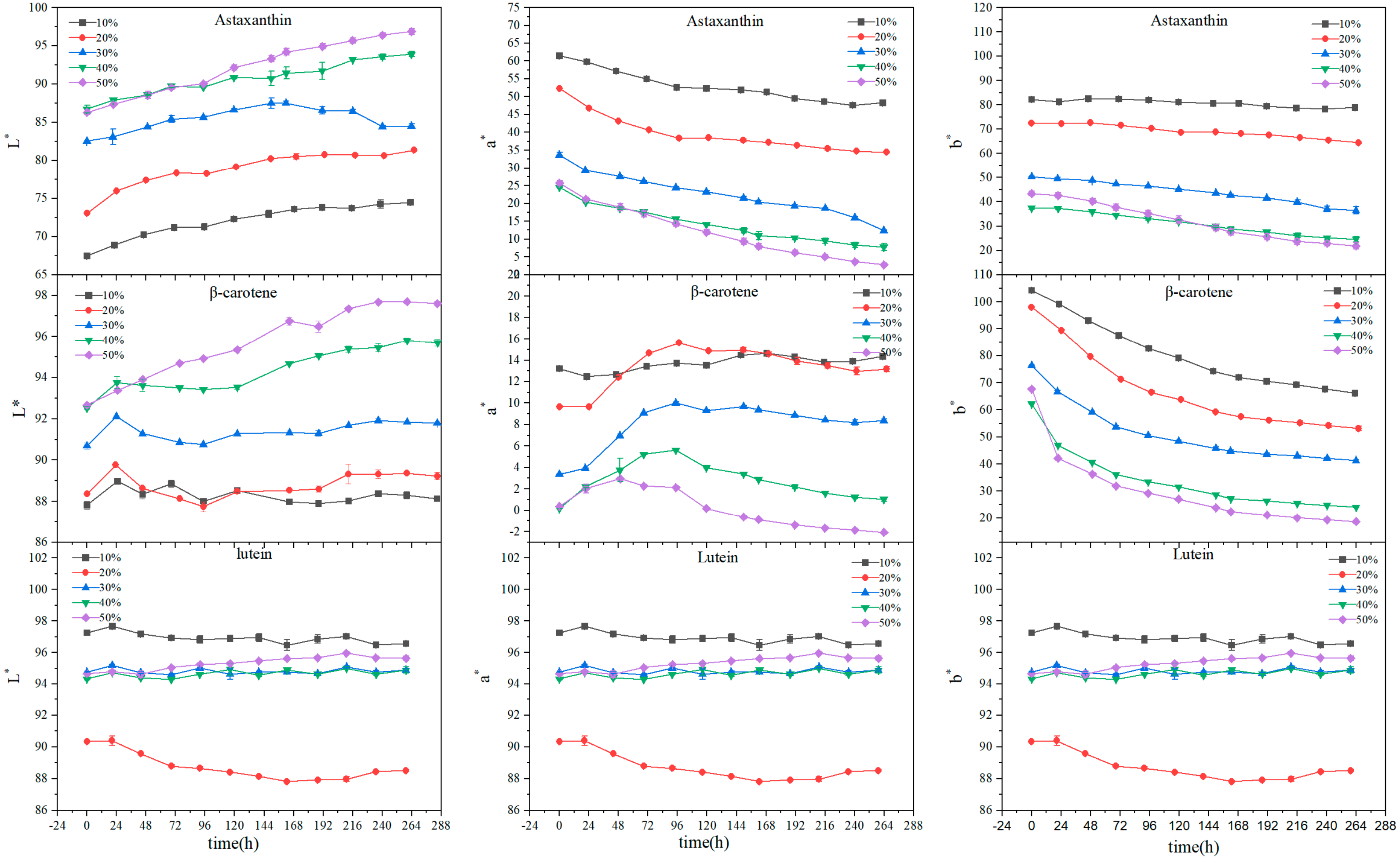


**Figure S3** Color parameters (L*, a* and b*) of [P4448]Cl aqueous solution containing astaxanthin (top), β-carotene (middle) and lutein (bottom) as a function of water content in storage at 25°C in the dark

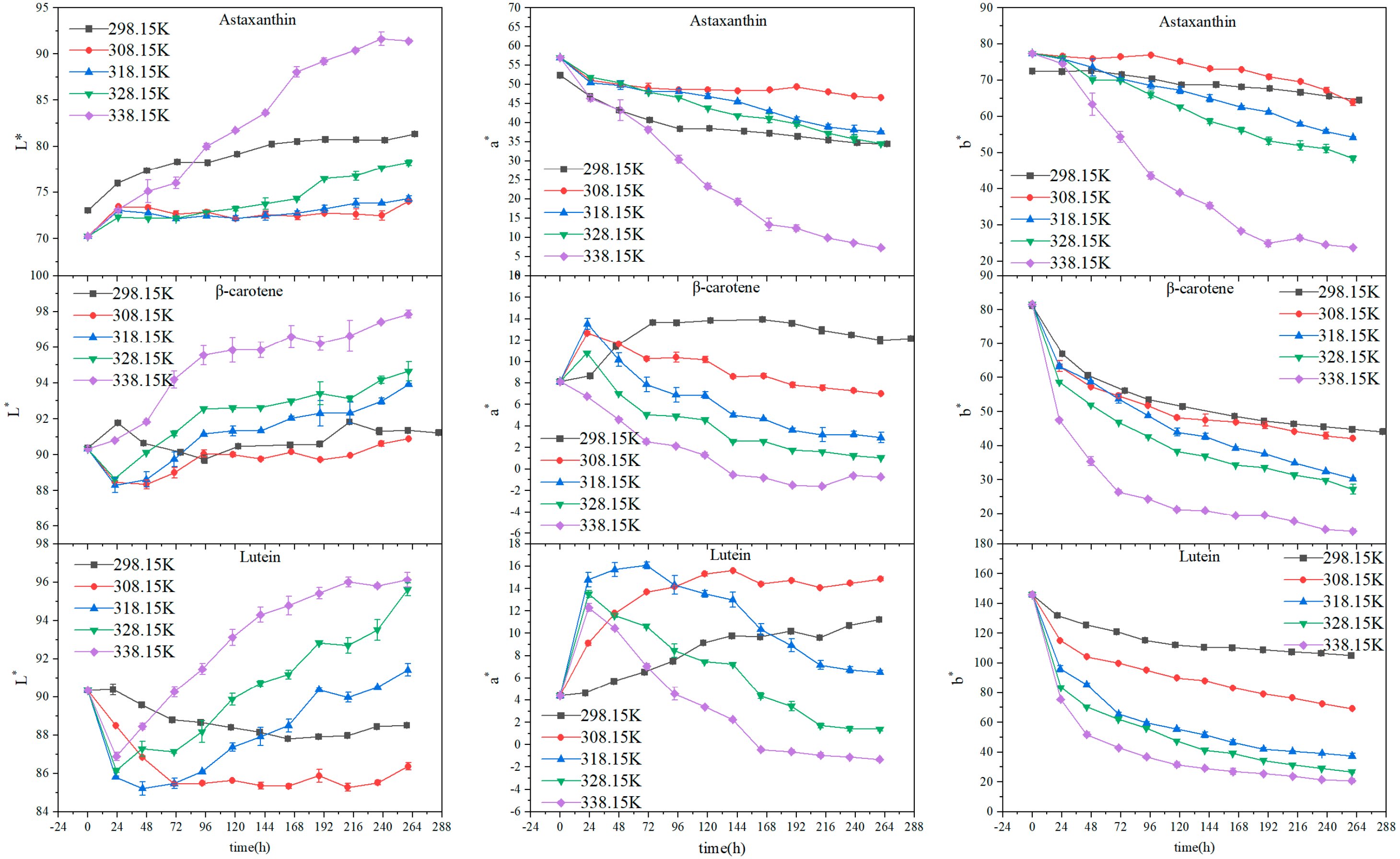


**Figure S4** Color parameters (L*, a* and b*) of [$P_{4448}$]Cl aqueous solution containing astaxanthin (top), β-carotene (middle) and lutein (bottom) as a function of temperature in storage at 20% water in the dark.

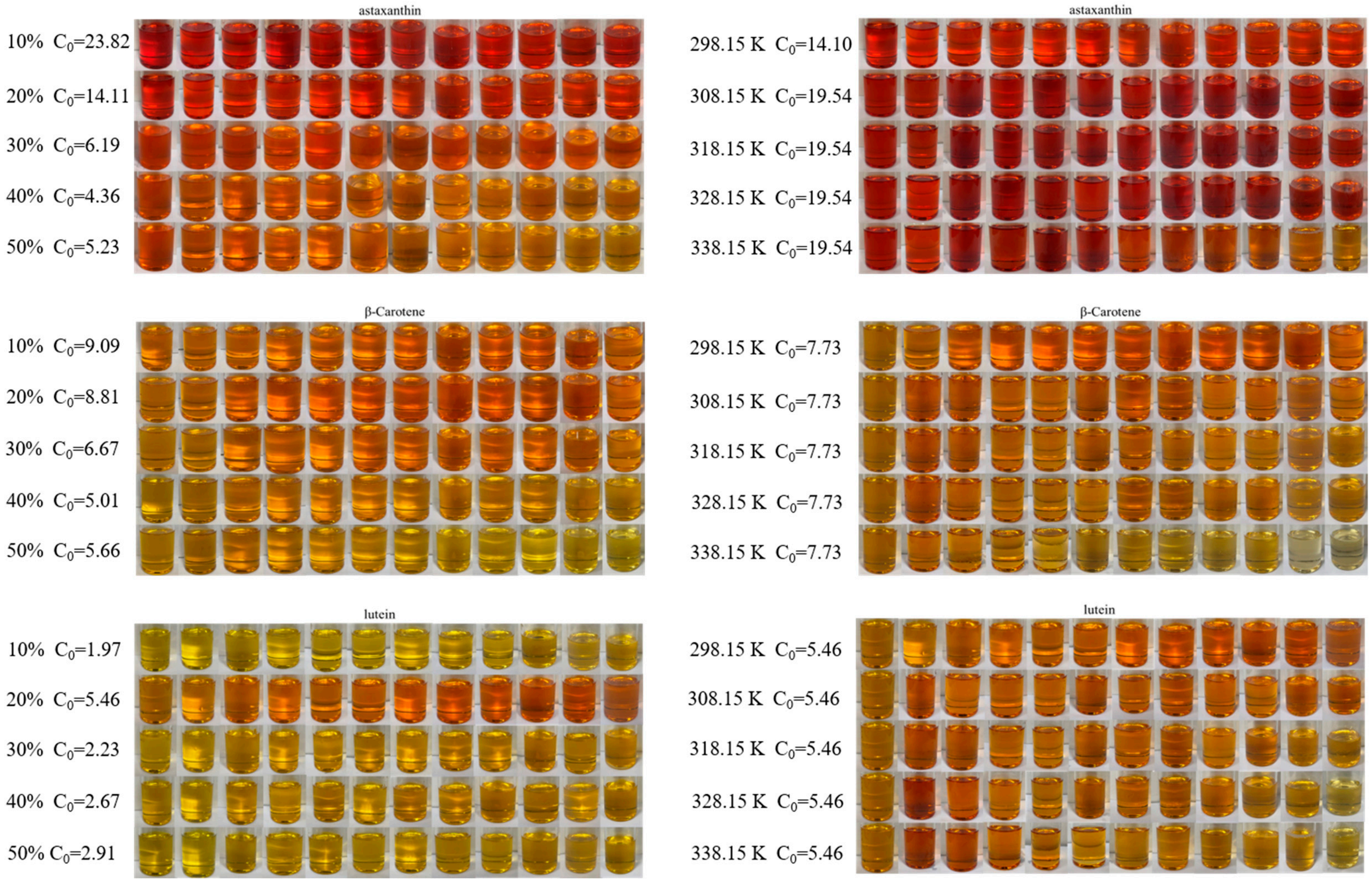


**Figure S5** Pictures of [$P_{4448}$]Cl aqueous solution containing astaxanthin (top), β-carotene (middle) and lutein (bottom) as a function of different storage conditions from 0 day to 12 day. $C_0$(μg/ml) represents the initial concentration of carotenoids in the solution.

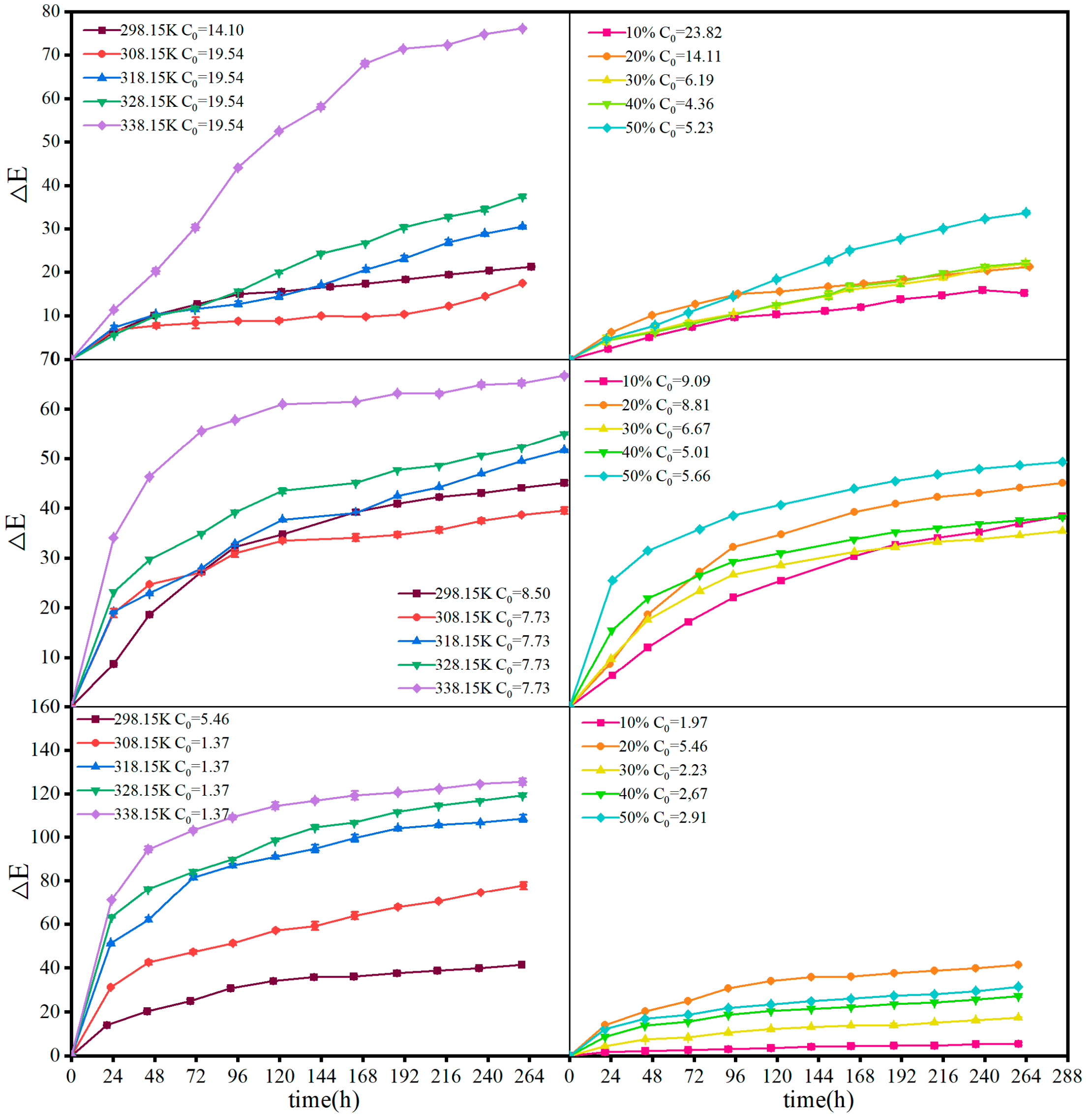


**Figure S6** ΔE of [$P_{4448}$]Cl aqueous solutions containing astaxanthin (top), β-carotene (middle), and lutein (bottom) in the storage for 12 days. $C_0$(μg/ml) represents the initial concentration of carotenoids in the solution.

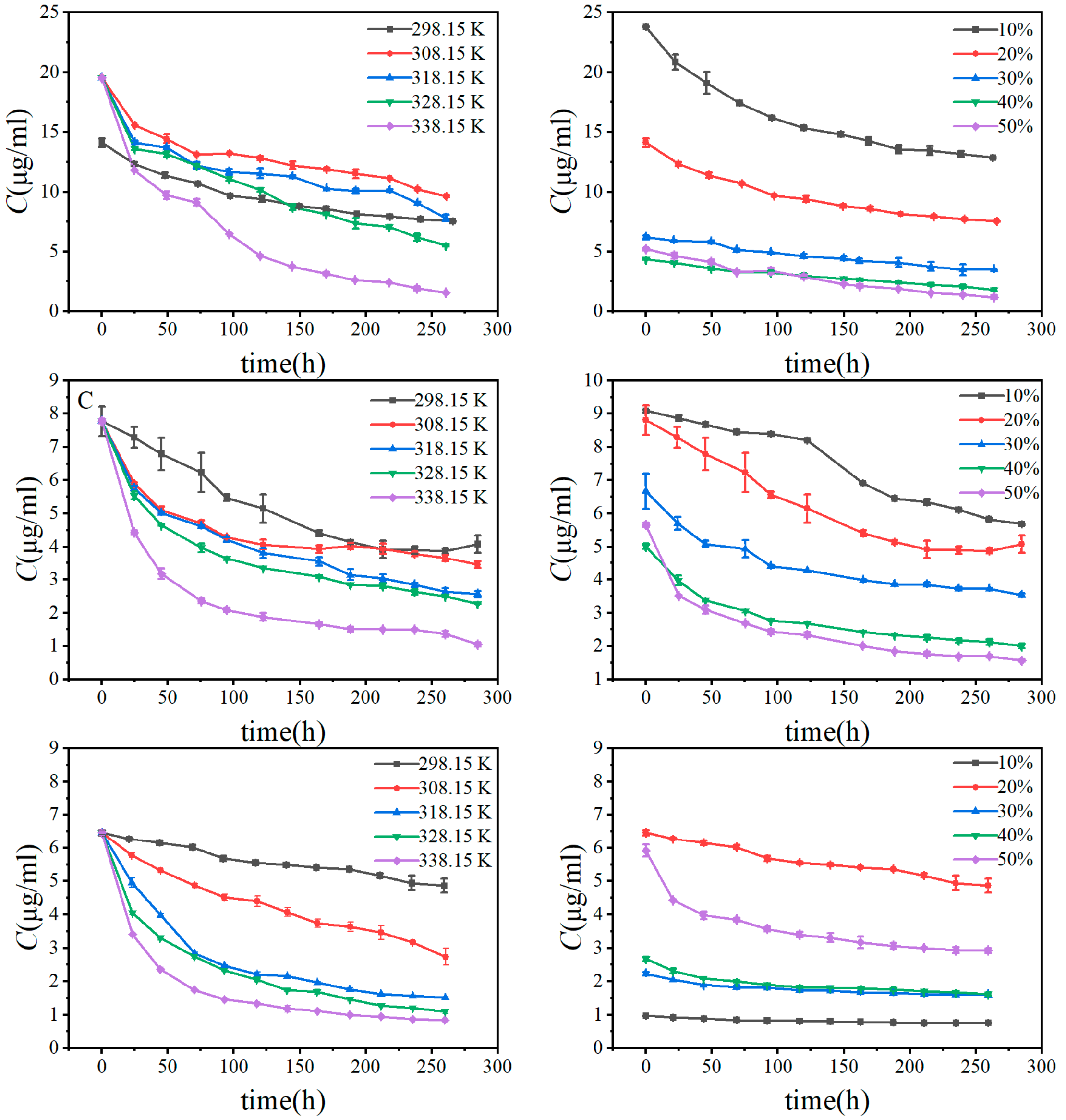


**Figure S7** Concentration($C$) of astaxanthin (top), β-carotene (middle), lutein (bottom) in $[P_{4448}]Cl$ aqueous solution in the storage for 12 days.
